# 5-methoxyindole metabolites of L-tryptophan: control of COX-2 expression, inflammation and tumorigenesis

**DOI:** 10.1186/1423-0127-21-17

**Published:** 2014-03-03

**Authors:** Kenneth K Wu, Huei-Hsuan Cheng, Tzu-Ching Chang

**Affiliations:** 1 Metabolomic Medicine Research Center, and Graduate Institutes of Basic and Clinical Medicine Science, China Medical University and Hospital, Taichung, Taiwan; 2 National Health Research Institutes, Zhunan, Taiwan; 3 National Tsing Hua University, Hsin-Chu, Taiwan

**Keywords:** Cyclooxygenase-2, Cytokine, Inflammation, Tumorigensis, Cancer metastasis, Melatonin, 5-methoxytryptophan, Cytoguardin, 5-hydroxytryptophan

## Abstract

Cyclooxygenase-2(COX-2) overexpression promotes inflammation and tumorigenesis. COX-2 expression in response to diverse stimuli is tightly controlled to avoid persistent overexpression. 5-methoxyindole metabolites of L-tryptophan represent a new class of compounds that control COX-2 expression at the transcriptional level. Two of the metabolites, the newly discovered 5-methoxytryptophan (5-MTP, also known as cytoguardin) and N-acetyl 5-methoxytryptamine (melatonin) are the focus of this review. 5-MTP is produced by mesenchymal cells such as fibroblasts via 5-hydroxytryptophan (5-HTP). It inhibits COX-2 transcriptional activation induced by diverse proinflammatory and mitogenic factors. Cancer cells are deficient in cytoguardin production which contributes to COX-2 overexpression. Fibroblast-generated 5-MTP is capable of restoring the control of COX-2 overexpression in cancer cells. 5-MTP blocks cancer cell migration and invasion in vitro and inhibits tumor growth and cancer metastasis in a xenograft model. Melatonin possesses similar COX-2 suppressing and anti-cancer properties albeit at supra-pharmacological concentrations. By contrast, 5-hydroxyindole metabolites of L-tryptophan such as 5-hydroxytryptamine (serotonin), 5-hydroxytryptophol and other serotonin catabolites do not control COX-2 expression. 5-hydroxytryptophan inhibits COX-2 expression through conversion to 5-MTP. The physiological relevance of 5-MTP as an endogenous regulator of inflammation and cancer metastasis remains to be investigated. On the other hand, 5-methoxyindole metabolites of tryptophan are valuable lead compounds for development of new anti-inflammatory drugs and cancer chemoprevention.

## Review

### Introduction

Cyclooxygenase-2 (COX-2, also known as Prostaglandin endoperoxide synthase-2 or PGH synthase-2; gene name is *PTGS-2*) is expressed in diverse cells notably inflammatory cells. At resting cellular state, expression of COX-2 is restricted to a very low level often barely detectable on the Western blotting. Upon cellular stimulation with cytokines, lipopolysaccharides (LPS), growth and mitogenic factors, there is a burst of expression of COX-2 resulting in robust production of prostaglandin G2 (PGG2) and PGH2 (PGG2 and PGH2 are collectively called PG endoperoxides) [1,2]. PGH2 is an intermediary metabolite in prostaglandin biosynthesis. It is converted to PGE2, PGF2α, PGD2, thromboxane A2 (TXA2) and prostacyclin (PGI2) by specific terminal enzymes i.e. PGE synthase, PGF synthase, PGD synthase, thromboxane synthase and PGI synthase, respectively. Production of PGs, TXA2 and/or PGI2 is governed by the level of the induced COX-2 and the expression of the downstream terminal enzymes. Prostaglandin production is cell-specific. For example, a majority of prostanoids produced by macrophages are PGE2 and TXA2, while the major prostanoid produced by vascular endothelial cells is PGI2. Selective prostanoid production in a cell-dependent manner permits the cells to carry out their unique physiological functions and participate in pathological processes. Since COX-2 occupies a key position in cell-selective prostanoid productions, it plays multiple physiological roles including vascular protection and reproduction, and mediates a number of important pathological conditions, notably inflammation and tumorigenesis. Persistent overexpression of COX-2 is of particular importance in mediating inflammatory tissue damages and cancer growth and metastasis. Persistent COX-2 overexpression is due to continuous and/or repeated stimulation of COX-2 expression in inflammatory cells by showers of proinflammatory cytokines or endotoxins. It could also be due to deregulation of COX-2 expression in cancer cells.

### Persistent stimulation of COX-2 expression in inflammatory cells

Macrophages are at the front line of tissue inflammation. In response to stimulation by immune mediators, endotoxins and cytokines, they express abundant COX-2 and generate PGE2 and TXA2 [3]. Tissue fibroblasts are also important inflammatory cells. At resting state, fibroblasts function as a supporter of tissue integrity. They lay down connective tissues and provide cellular support for tissues. However, upon tissues injuries, they migrate to the injured sites where they change to a proinflammatory phenotype [4]. In response to exogenous stimuli, they express robust COX-2 and generate abundant PGE2 to mediate tissue inflammation [5,6].

Proinflammatory mediators stimulate COX-2 transcription in diverse cell types such as macrophages, fibroblasts and endothelial cells by a common mechanism. Upon stimulation by LPS, IL-1β, TNFα or PMA, human fibroblasts exhibit enhanced binding of NF-κB(p65/p50), C/EBPβ, AP-1 and CREB-2/ATF2 concurrently to their respective cognitive sites on COX-2 promoter/enhancer region within 500-bp from the transcription start sites [7-9]. Furthermore, p300 binding to the enhancer sites is correspondingly increased [10,11]. Proinflammatory mediators stimulate P300 histone acetyltransferase (HAT) activity which in turn augments transactivators binding by acetylation of chromatin histone and the transactivators (p65, C-Jun, C/EBPβ and CREB-2)[12]. Prolonged continuous and/or repeated stimulation by exogenous insults has been shown to result in persistent overexpression of COX-2 and excessive production of proinflammatory prostaglandins in fibroblasts, macrophages or endothelial cells. COX-2 overexpression is considered to play a major role in human inflammatory disorders such as rheumatoid arthritis and degenerative joint diseases as evidenced by effective control of joint inflammation by selective COX-2 inhibitors [13,14].

### Deregulation of COX-2 expression in cancer cells

There is ample evidence that COX-2 is constitutively overexpressed in cancer cells [15]. Malignant tumors are skillful in recruiting inflammatory, immune and vascular cells to create an inflammatory microenvironment, where proinflammatory cytokines released by macrophages, fibroblasts and endothelial cells stimulate cancer COX-2 expression. Furthermore, cancer cells produce growth factors to stimulate COX-2 expression in the inflammatory and stromal cells within the tumor microenvironment [16]. The positive regulatory loop leads to persistent COX-2 overexpression not only in cancer cells but also in the microenvironmental stromal and inflammatory cells. This results in robust production of PGE2 and other prostanoids which promote cancer growth and metastasis.

It was demonstrated that COX-2 overexpression alters cell phenotypes, which are characterized by reduced response to apoptotic signals, increased cellular migratory activity and through production of matrix metalloproteinases (MMPs), increased cell invasiveness [17,18]. Thus, COX-2 overexpression in cancer cells and cancer microenvironment represents a major force for tumor growth and cancer metastasis. The causal role of COX-2 overexpression in tumorigenesis is supported by strong clinical and experimental evidence. Selective small-molecule inhibitors of COX-2 catalytic activity (coxibs), prevent growth of carcinogen-induced cancer in experimental animals or cancer induced by genetic modification [19-21].

The exact mechanism by which COX-2 is constitutively overexpressed in cancer cells is not entirely clear. There are suggestions that aberrant Wnt/β-catenin signaling due to mutation of APC (adenomatous polyposis coli), β-catenin, or glycogen synthase Kinase 3 (GSK3) may be a common mechanism by which COX-2 expression is enhanced in gastrointestinal cancers, notably colorectal cancer [22-25]. Under physiological conditions in normal cells, β-catenin is sequestered and degraded in a destruction complex composed of APC, GSK-3, axin and casein kinase 2(CK2). Loss of function and mutation of APC in colorectal cancer or GSK-3 in gastric cancer disrupts the function of the destruction complex and liberates β-catenin. β-catenin enters nucleus where it forms a complex with Tcf(T cell factor), binds to the promoter region and activates the transcription of a number of genes important in cell growth, and tumorigenesis. It was reported that β-catenin/Tcf binds to the promoter region of COX-2 and enhances COX-2 expression [26]. Other signaling and transcriptional pathways are likely to be dysregulated in different cancers. Further investigations are necessary to discern the dysregulated mechanism for each type of cancer.

COX-2 in cancer cells and the microenvironmental stromal cells catalyze the formation of several prostanoids among which PGE2 is considered to be the most important in promoting cancer cell growth and metastasis [27]. PGE2 is pleiotropic and promotes cancer cell proliferation and tumor growth by several mechanisms [28,29]. A key signaling pathway via which PGE2 induces cancer growth is Wnt/β-catenin pathway [30,31]. PGE2 binds to EP2 receptor and induces dissociation of Gs into βγ and α subunits. Gs βγ subunit activates PI-3 K/Akt. Akt inhibits GSK-3β resulting in release of β-catenin from the APC-GSK-3β-axincomplex. Under hypoxic conditions, β-catenin complexes with hypoxia inducing factor 1 (HIF-1) which drives the expression of vascular endothelial growth factor (VEGF) to enhance angiogenesis. The α subunit interacts with axin of the APC-GSK-3β-axin complex which triggers release of β-catenin, further enhancing the expression of proliferative and angiogenic genes. Gsβγ subunit also activates Ras/Raf/Mek/Erk pathway resulting in enhanced expression of Bcl-2, conferring resistance to apoptosis.

PGE2 stimulates cancer cell migration and invasion by inducing cancer cell cytoskeletal reorganization, production of MMPs and epithelial-mesenchymal transition (EMT) [32-35]. The actions of PGE2 on cell proliferation, resistance to apoptosis, migration and invasion contribute to the effect of COX-2 overexpression on cancer cells. It may be concluded that through PGE2 productions, COX-2 overexpression plays a critical role in promoting cancer growth and metastasis.

### Control of COX-2 expression by 5-methoxytryptophan

For a long time it has been a curious question whether COX-2 expression under inflammatory challenge is protected by endogenous factors. Hence, we were intrigued when we serendipitously identified COX-2 suppressing activity in the conditioned medium (CM) of cultured human foreskin fibroblasts, Hs68 [36]. Chemical analysis of semi-purified fractions of CM revealed that the COX-2 suppressing activities reside in the small molecule fraction (Mr < 500 daltons). NMR analysis suggested presence of indoles in the active molecular fraction. The COX-2 suppressing activity in the CM fraction was named cytoguardin [36].

**Figure 1:**
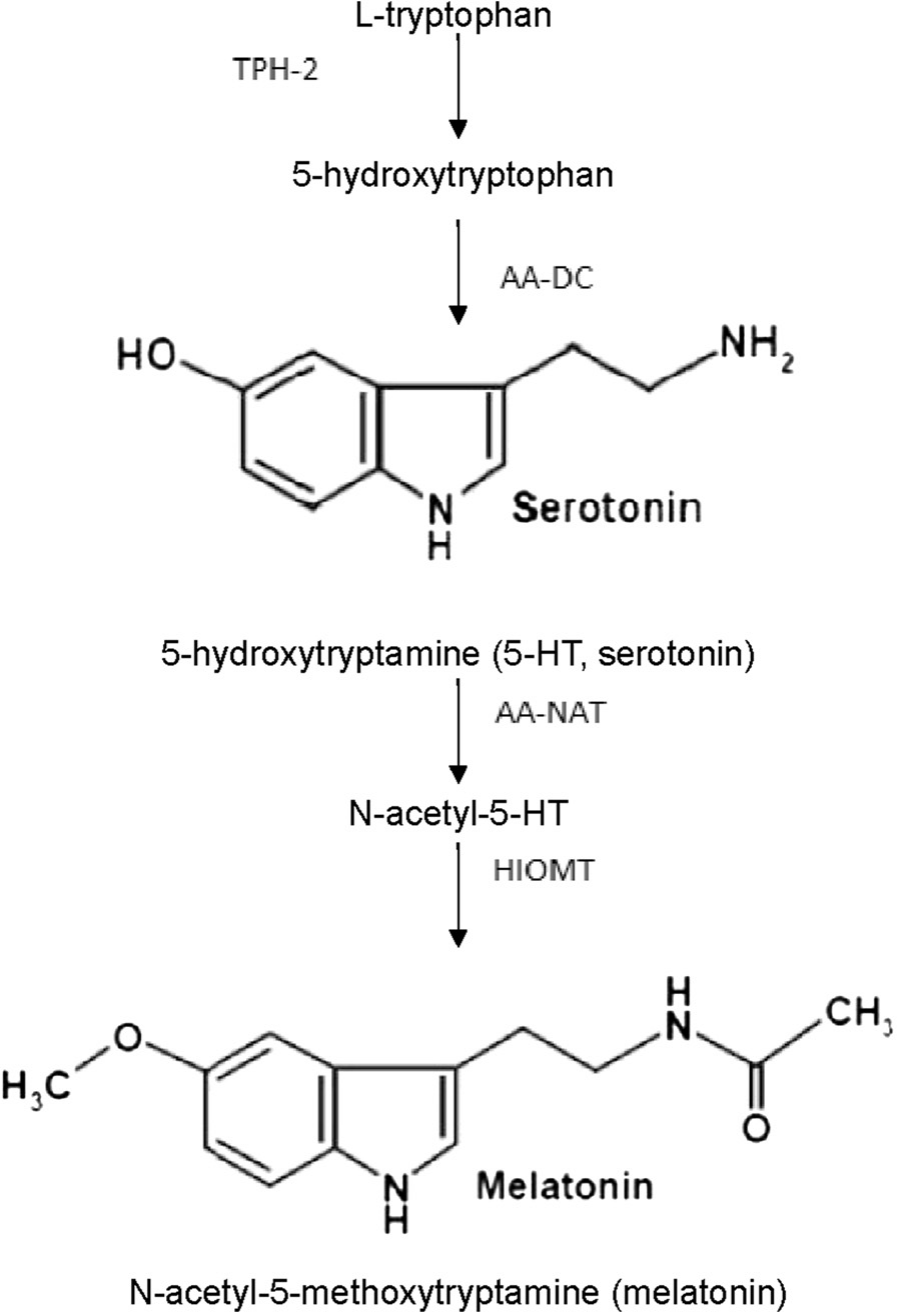
**Melatonin synthesis.** Abbreviations are: TPH-2, Tryptophan hydroxylase-2; AA – DC, aromatic aminoacid decarboxylase; AA-NAT, arylalkylamine N-acetyltransferase; HIOMT, hydroxyindole O-methyltransferase.

The chemical nature of cytoguardin was recently identified by comparative metabolomics. Based on the observations that cancer cells do not release detectable cytoguardin activity in the conditioned medium, metabolomic profiles of purified fraction of fibroblasts CM vs that of A549 lung cancer cell CM were obtained by ultrahigh performance liquid chromatography (UPLC) coupled to quadruple time of flight (Q Tof) mass spectrometry(MS). Analysis of the spectra of chromatography and mass spectrometry reveals striking differences. The fibroblast CM contains several m/z peaks which are barely detectable in A549 CM [37]. Two major peaks, m/z 276.1 and m/z 262.1 were selected for more detailed analysis. Through chemical database search and biochemical deductions, m/z 262.1 was determined to be a known compound, i.e. 5-hydroxytryptophan (5-HTP). 5-HTP is a key intermediate metabolite of serotonin biosynthesis and melatonin biosynthesis. In neuronal cells, L-tryptophan is converted to 5-HTP by tryptophan hydroxylase-2 (TPH-2) and 5-HTP is converted to 5-hydroxytryptamine (serotonin, 5-HT), by aromatic amino acid decarboxylase [38]. In pineal cells, 5-hydroxtryptamine is further converted to N-acetyl-5-HT by arylalkylamine N-acetyltransferase (AA-NAT) and N-acetyl-5-HT is converted to melatonin (N-acetyl-5-methoxytryptamine) by hydroxyindole-O-methyltransferase (HIOMT) [39] (Figure 1). Interestingly, addition of 5-HTP to fibroblasts enhanced COX-2 suppressing activity and increased the m/z 276.1 peak suggesting that 5-HTP is converted to m/z 276.1. On the chemical database, one of the candidate molecules for m/z 276.1 is 5-methoxytryptophan (5-MTP). As little is known about 5-MTP production by mammalian cells, 5-MTP production by fibroblasts must be validated. Analysis of fibroblast CM by 5-MTP specific enzyme immunoassay confirmed the presence of abundant 5-MTP. By contrast, 5-MTP in A549 CM was barely detectable and not significantly different from that in serum-containing culture medium. Importantly, addition of 5-HTP to Hs68 fibroblasts resulted in elevation of 5-MTP level in CM [37]. On the other hand, 5-HTP did not boost 5-MTP in cancer cells.

**Figure 2:**
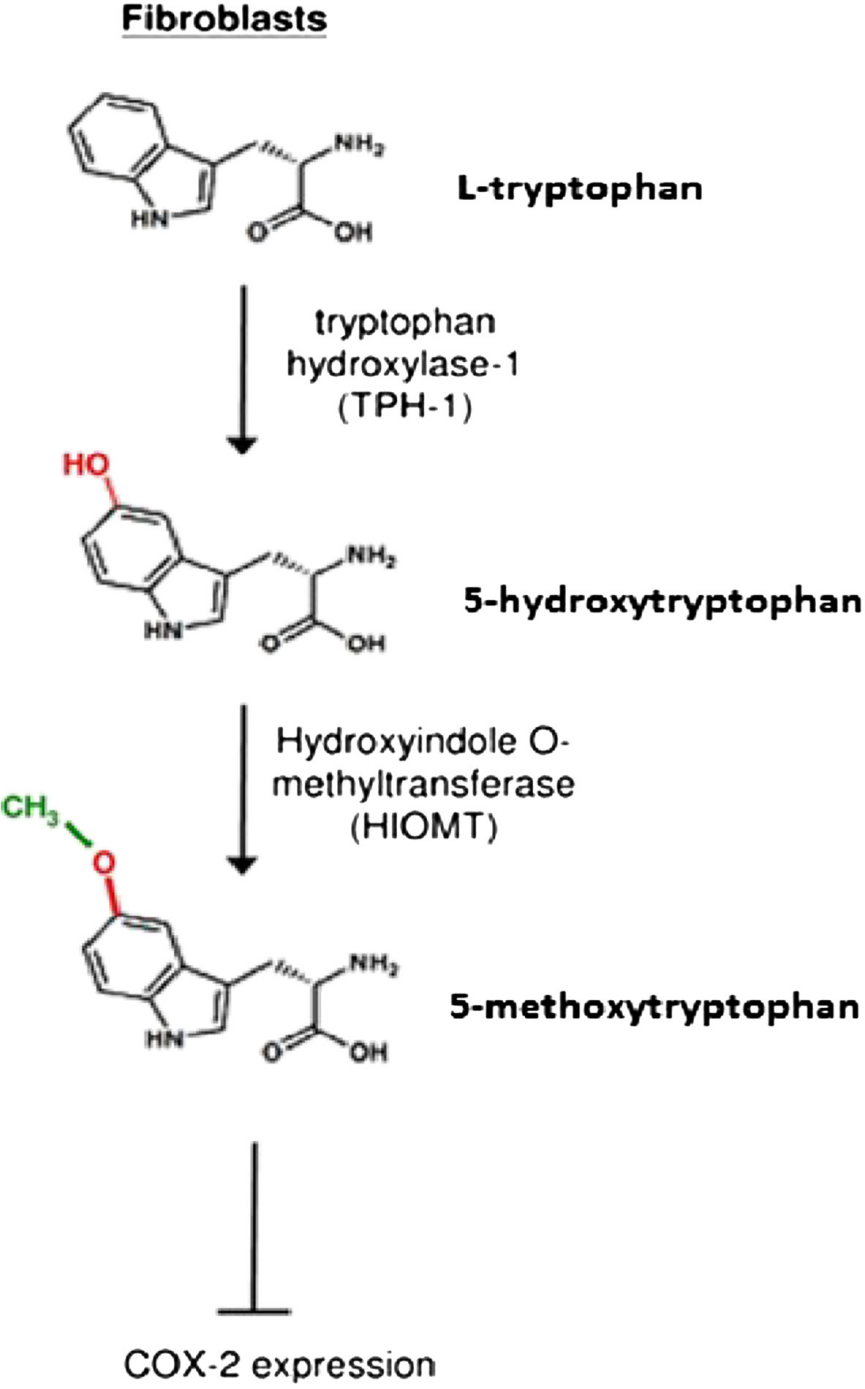
Cytoguardin (5-MTP) biosynthesis in human fibroblasts.

Since 5-MTP is structurally similar to melatonin, it was reasoned that 5-MTP could be converted from 5-HTP by HIOMT. This notion was supported by the expression of HIOMT proteins in Hs68 cells. Importantly, silencing of HIOMT with selective siRNA resulted in aborted 5-MTP in the CM and abrogation of COX-2 suppression [37]. Hs68 fibroblasts express TPH-1 isoform and silencing of TPH-1 with siRNA also abolished 5-MTP production and COX-2 suppression. Addition of 5-HTP rescued 5-MTP production and COX-2 suppression. Taken together, these results demonstrate that fibroblasts possess functional TPH-1 and HIOMT enzymes to synthesize 5-MTP which is released to extracellular milieu to act as an autacoid to control COX-2 expression. A tentative 5-MTP synthetic pathway is illustrated in Figure 2.

### Cytoguardin restores cancer cell COX-2 suppressing activity

Constitutive COX-2 overexpression in cancer-cells is attributed to uncontrolled transcriptional activation by growth factors and cytokines. Since cancer cells fail to release cytoguardin, defective 5-MTP production may contribute to COX-2 overexpression. In this case, CM from fibroblasts should be able to suppress cancer COX-2 expression. This was proved by co-cultures of Hs68 with A549 cells in a Boyden Chamber. Hs68 at sufficient cell numbers were effective in suppressing A549 COX-2 expression. Direct incubation of A549 cells with Hs68 fibroblast CM exerted comparable dose-dependent effect. 5-MTP exerted a concentration-dependent inhibition of A549 COX-2 expression. 5-MTP at 10 μM significantly suppresses COX-2 expression and maximally inhibits COX-2 expression at 50–100 μM [37].

### 5-MTP inhibits cancer cell migration/invasion and cancer growth and metastasis

Since COX-2 overexpression promotes cancer cell proliferation and migration, it was stipulated that 5-MTP, through its suppression of COX-2 expression is capable of blocking cancer growth and metastasis. Indeed, addition of 5-MTP to the cultured medium of A549 cells resulted in reduction of A549 migration as analyzed in a transwell assay [37]. Furthermore, 5-MTP inhibited A549 invasion through matrigel in the transwell assay [37]. Concentrations of 5-MTP required for inhibition of migration were corresponding to those needed to inhibit invasion which were correlated with COX-2 suppression [37]. Thus, it is likely that 5-MTP inhibits cancer cell migration and invasion through blocking COX-2 transcriptional activation.

5-MTP possesses anti-cancer effects in vivo. Intraperitoneal injection of 5-MTP into a murine A549 xenograft tumor model resulted in a time-dependent reduction of cancer growth. Tumor volume at 7 weeks after 5-MTP administration was ~50% of that of vehicle control. 5-MTP also significantly reduced metastatic lung nodules [37]. These results support the notion that 5-MTP suppresses cancer growth and metastasis through inhibition of COX-2 overexpression.

### Control of COX-2 expression by other 5-HTP metabolites

A large number of 5-HTP metabolites have been identified, among which serotonin and melatonin are of physiological important. Serotonin is catabolized to form 5-hydroxyindole compounds notably 5-hydroxyindole acetic acid, and 5-hydroxytryptophol. It is interesting to note that except for 5-HTP, none of the 5-hydroxyindole catabolites of serotonin is active in suppressing COX-2 expression [37]. Serotonin also does not possess COX-2 suppressing action at concentrations up to 1 mM [40]. By contrtast, 5-methoxyindole metabolite such as melatonin inhibits COX-2 expression at millimolar concentrations. These results suggest that the 5-methoxyindole moiety is important in controlling COX-2 expression.

### Melatonin suppresses COX-2 expression

Melatonin is produced primarily in pineal gland by pinealocytes, and is involved in regulating sleep and other biological activities associated with circadian rhythm [41]. Interestingly, several studies have reported that melatonin possesses COX-2 suppressing actions. Melatonin at supra-pharmacological concentrations (≧1 mM) was reported to inhibit proinflammatory mediator-induced COX-2 expression at the transcriptional level [40,42]. Melatonin was also reported to control COX-2 expression in colitis in a rat model [43]. As the blood melatonin concentrations are generally less than 1nM, the physiological role that melatonin plays in controlling COX-2 and COX-2 mediated pathophysiological processes is questionable. However, recent studies report that macrophages produce melatonin in response to stimulation by LPS, cytokines and PMA [44] and it was suggested that tissue injuries such as ischemia-reperfusion injury stimulates inflammatory cells to generate abundant melatonin [45]. As the local melatonin concentration has not been determined, it remains to be investigated whether melatonin may be a functionally important local hormone to defend against excessive inflammation and tissue damage.

**Figure 3:**
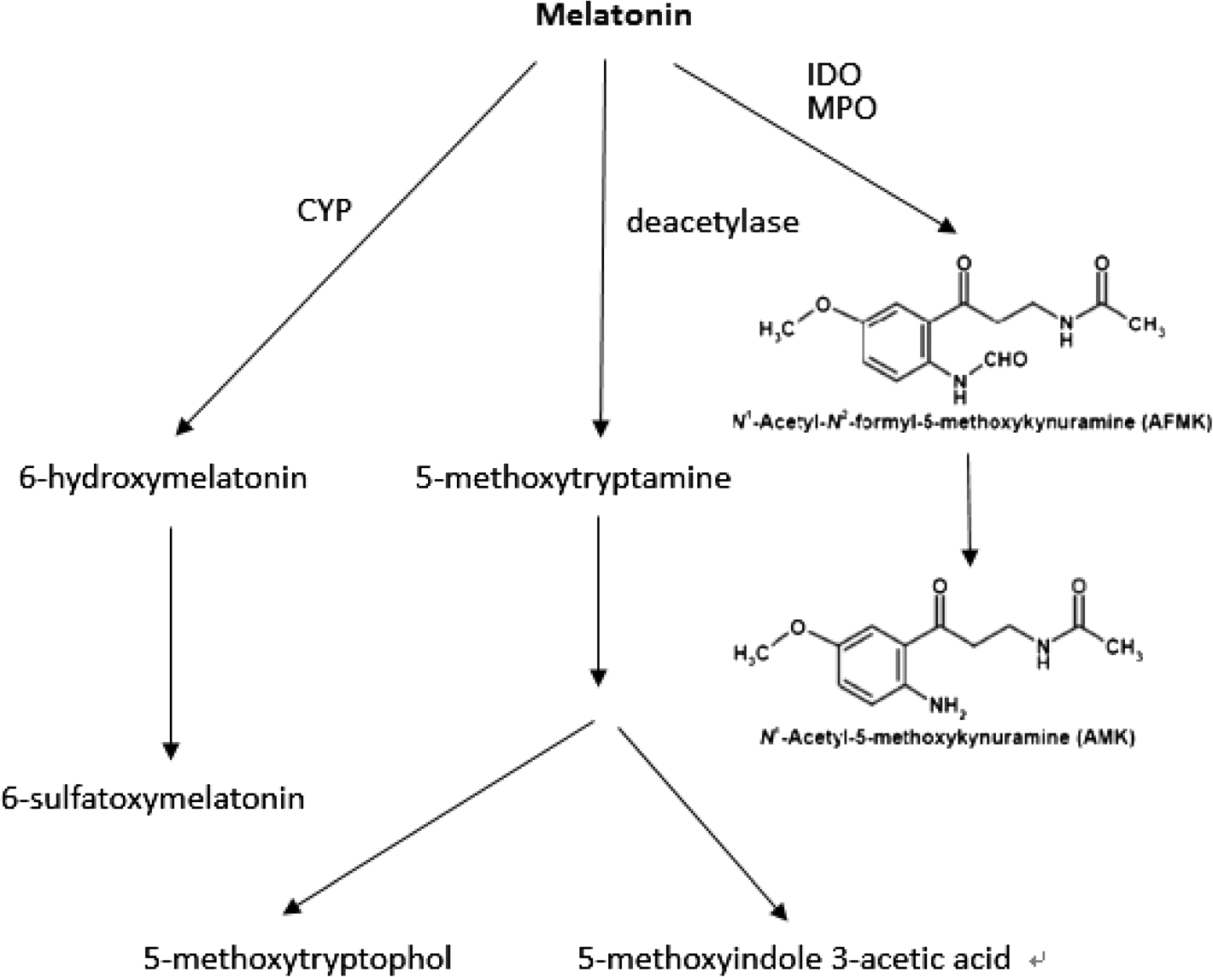
**Major pathways of melatonin catabolism.** CYP denotes cytochrome p450 1A2, 1A1, 1B1; MPO, myeloperoxidase; IDO, indoleamine 2, 3-dioxygenase.

Melatonin is catabolically converted to indole compounds via three major pathways: (1) degradation by cytochrome p450s i.e. CYP1A2, CYP1A1 and CYP1B1 to 6-hydroxy-melatonin which is further converted to 6-sulfatoxy-melatonin by a sulfotransferase; (2) conversion to N^1^ acetyl-N^2^ formyl 5- methoxykynuramine (AFMK) by myeloperoxidase or indoleamine 2, 3 dioxygenase (IDO) which is further converted to N^1^ acetyl-5-methoxykynuramine (AMK) by formamidases; (3) Deacetylation to form 5-methoxytryptamine (5-MT) [46] (Figure 3). Two of the 5-methoxyindole metabolites, i.e. AFMK and AMK were reported to inhibit COX-2 expression in macrophages [42].

The inhibitory effects of melatonin on cancer cell proliferation have been extensively investigated. Melatonin reduces proliferation of several types of cancer cells, notably MCF7 breast cancer cells. Early reports indicate that melatonin at the physiological concentration (<1 nM) inhibited MCF-1 cell proliferation [47]. However, subsequent studies failed to confirm suppression of MCF-1 proliferation by melatonin at the low concentrations. Others have reported the inhibition of MCF-7 proliferation by melatonin at millimolar concentrations [48]. The reasons for the discrepancy are unclear but could be attributed to variations in experimental conditions and variations of MCF-7 cells [49]. Melatonin at ≧1 mM inhibits MDA-MB-361 cancer cell proliferation and induces apoptosis which is correlated with suppression of COX-2 expression and PGE2 production [50].

The effect of melatonin on cancer growth has also been evaluated in animal models and the results are inconsistent [51]. Variations in the in vivo data, like those in the in vitro cell studies are influenced by melatonin dosing. Furthermore, melatonin in circulation is rapidly degraded into several catabolites some of which may possess oncostatic activity. Length of treatment may be another confounding factor influencing the effect of melatonin.

### Therapeutic potentials of 5-methoxyindole metabolites

5-methoxyindole compounds, notably cytoguardin (5-MTP) and melatonin have potentials as a therapeutic agent for inflammatory disorders and as a cancer chemopreventive agent. As these compounds are endogenously produced, they have the advantage of having predictable adverse effects as long as they are not too excessive. They are different from the selective COX-2 inhibitors in that they only block COX-2 overexpression induced by proinflammatory mediators and mitogenic factors. As they do not inhibit the basal vascular endothelial cell COX-2, as COX-2 inhibitors do, they will avoid the cardiovascular complications of selective COX-2 inhibitors [52].

A number of clinical trials have been carried out to evaluate the efficacy of melatonin in treating cancer. The results are inconsistent, which may be attributed to small patient numbers and variable dosing. Interestingly, meta-analysis of 21 clinical trials revealed a beneficial effect of melatonin on one-year survival and tumor response. Furthermore, it reduces the serious adverse effects of concomitant chemotherapy [53]. Large randomized control trials are needed to validate the efficacy of melatonin in treating human cancers.

An attractive idea is emerging regarding the use of melatonin as an adjuvant to enhance the efficacy of conventional cancer chemotherapy or biotherapy [53]. This idea has not been supported by solid clinical trials results and will require further investigation.

Cytoguardin is a promising lead compound for therapy of a broad spectrum of inflammation-related diseases including cancer chemoprevention. A major advantage is that the effective concentrations of 5-MTP for control of cancer growth and metastasis in vitro and in vivo are in line with the normal human serum concentrations (unpublished data).

## Conclusion

L-tryptophan, in addition to being an essential amino acid for protein synthesis, serves as an important precursor molecule for production of active metabolites involved in regulation of immunity, inflammation, neurotransmission and circadian rhythm. Those biologically active metabolites are produced via two major pathways: (1) indoleamine 2, 3 dioxygenase (IDO) pathway which generates kynurenines, and (2) tryptophan hydroxylase (TPH) pathway which was originally thought to produce neurotransmitters and neural function modulators notably serotonin and melatonin. Discovery of 5-MTP pathway adds a new dimension to the functional diversity of tryptophan and complexity of tryptophan metabolism.

The TPH pathway generates a series of 5-methoxyindole metabolites whose synthetic and catabolic pathways are not fully understood. Take 5-MTP as an example, the synthetic pathway may not have been completely uncovered. There may be intermediary steps to be further discovered. It is of interests to note that 5-MTP synthesis shares with melatonin synthesis common enzymatic steps, i.e. TPH and HIOMT. However, a striking difference between 5-MTP and melatonin biosynthesis is the decarboxylation step which is required for melatonin but not for 5-MTP production. Metabolomics analysis of fibroblast CM detects only 5-methoxyindole derivatives of 5-MTP but not melatonin or its catabolites. The results imply that 5-MTP vs. melatonin production is cell type-specific. Pineal, retinal and enteroendocrine cells are equipped with enzymes to produce melatonin while peripheral non-neural cells such as fibroblasts and vascular cells are endowed with enzymes to catalyze the formation of cytoguardin. Melatonin and its metabolites are primarily involved in regulating brain functions whereas cytoguardin and its metabolites control inflammatory responses and tumorigenesis.

Melatonin was reported to inhibit p300 HAT and NF-κB activation induced by proinflammatory mediators [40,50]. Unpublished data reveal that 5-MTP inhibits p300 HAT activation at concentrations comparable to its inhibition of COX-2 expression. These results suggest that 5-methoxyindole metabolites target at least in part p300 HAT activation and NF-κB and consequently blocks the transactivation of not only COX-2 but also inducible nitric oxide synthase [40,43] and proinflammatory cytokines.

5-methoxyindole metabolites of L-tryptophan encompass a novel class of endogenously produced compounds to modulate the inflammatory response to environmental insults and control neoplastic growth and cancer metastasis. A critical issue regarding the physiological role of 5-methoxyindole metabolites, notably 5-MTP is whether under pathophysiological conditions, 5-MTP is produced in sufficient concentrations to combat excessive inflammatory reactions and tumorigenesis. Relevant to this issue is whether 5-MTP acts as a circulating hormone or as a local autacoid. Additional work is needed to resolve this issue. On the other hand, there seems little doubt that 5-MTP has a great potential to be considered as a candidate drug or as a lead compound for synthesizing new agents to treat or prevent diverse human inflammatory disorders and cancers.

## Competing interests

The authors declare that they have no competing interests.

## Authors’ contributions

KKW drafted, revised and approved the final version of the review article; HHC drafted and approved the final version; TCC drafted and approved the final version. All authors read and approved the final manuscript.

## Authors’ information

Dr. Kenneth K. Wu is distinguished professor and Director, Metabolomic Medicine Research Center, China Medical University and Hospital, Taichung Taiwan, and holds JD Ho Endowed Chair, National Tsing Hua University, Hsin-Chu, Taiwan. Dr. Huei-Hsuan Cheng and Dr. Tzu-Ching Chang are assistant professor, Graduate Institute of Clinical Medicine Science, China Medical University.
